# A Deep Learning-Based Approach for Identifying the Medicinal Uses of Plant-Derived Natural Compounds

**DOI:** 10.3389/fphar.2020.584875

**Published:** 2020-11-30

**Authors:** Sunyong Yoo, Hyung Chae Yang, Seongyeong Lee, Jaewook Shin, Seyoung Min, Eunjoo Lee, Minkeun Song, Doheon Lee

**Affiliations:** ^1^ School of Electronics and Computer Engineering, Chonnam National University, Gwangju, South Korea; ^2^ Department of Otorhinolaryngology-Head and Neck Surgery, Chonnam National University Medical School and Chonnam National University Hospital, Gwangju, South Korea; ^3^ Big Data Steering Department, National Health Insurance Service, Wonju, South Korea; ^4^ Department of Physical and Rehabilitation Medicine, Research Institute of Medical Science, Cardiovascular Research Institute, Chonnam National University Medical School and Hospital, Gwangju, South Korea; ^5^ Bio-Synergy Research Center, Daejeon, South Korea; ^6^ Department of Bio and Brain Engineering, Korea Advanced Institute of Science and Technology (KAIST), Daejeon, South Korea

**Keywords:** natural compound, natural product, medicinal use, deep learning, molecular interaction, chemical property, network analysis, text mining

## Abstract

Medicinal plants and their extracts have been used as important sources for drug discovery. In particular, plant-derived natural compounds, including phytochemicals, antioxidants, vitamins, and minerals, are gaining attention as they promote health and prevent disease. Although several *in vitro* methods have been developed to confirm the biological activities of natural compounds, there is still considerable room to reduce time and cost. To overcome these limitations, several *in silico* methods have been proposed for conducting large-scale analysis, but they are still limited in terms of dealing with incomplete and heterogeneous natural compound data. Here, we propose a deep learning-based approach to identify the medicinal uses of natural compounds by exploiting massive and heterogeneous drug and natural compound data. The rationale behind this approach is that deep learning can effectively utilize heterogeneous features to alleviate incomplete information. Based on latent knowledge, molecular interactions, and chemical property features, we generated 686 dimensional features for 4,507 natural compounds and 2,882 approved and investigational drugs. The deep learning model was trained using the generated features and verified drug indication information. When the features of natural compounds were applied as input to the trained model, potential efficacies were successfully predicted with high accuracy, sensitivity, and specificity.

## Introduction

A large number of medicinal plants possess diverse natural compounds, contributing to drug development by providing novel candidate therapeutic agents against various diseases. Natural compounds are small molecules synthesized by living organisms, including primary and secondary metabolites (Hanson, 2003). Accumulating evidence has shown that the ingestion of bioactive natural compounds, such as phytochemicals, antioxidants, vitamins, and minerals, through a diet rich in herbs, fruits, vegetables, and spices may promote health via negative immunoregulatory and anti-inflammatory activities (Chu et al., 2002; Mursu et al., 2013; Kruk, 2014). Moreover, many natural compounds have been proven to play an important role as modulators of cell signaling and homeostasis, which enforces the need to identify the medicinal potentials of bioactive natural compounds (Brindha, 2016; Dias et al., 2016; Pellavio et al., 2017).

Most previous studies on the identification of the medicinal uses of natural compounds used *in vitro* assessments (Foster et al., 2001; Iacopini et al., 2008; Li et al., 2008). In these studies, *in vitro* screening tests were performed for the assessment of the biological activities of natural compounds. However, large-scale experiments are needed as the number of considered natural compounds and candidate effects increases, which exponentially increases time and cost. Therefore, *in silico* approaches, which mostly focus on specific information such as molecular properties, chemical similarities, or clinical knowledge, have been proposed to predict medicinal candidates from natural compounds. Molecular-based approaches focus on finding similar responses or mechanisms between natural compounds and drugs from various networks, e.g., functional protein interactions or compound-target interactions (Tao et al., 2013; Kibble et al., 2015; Rampogu and Rampogu Lemuel, 2016). Chemical-based approaches investigate bioactive natural compound candidates by examining physicochemical properties and physiological effects (Zhou et al., 2010; Chen et al., 2017; Muhamad et al., 2017). However, the molecular targets, mechanisms, and chemical structure information of natural compounds are largely hidden, compared with those of approved drugs (Sutter and Wang, 1993; Lee, 1999; Yoo et al., 2018c). Therefore, both molecular and chemical-based approaches have low coverage and usability. Knowledge-based approaches apply statistical analysis to scientific databases, such as PubMed, or clinical trial information to identify medicinal natural compound candidates for a certain disease (Butler, 2005; Jensen et al., 2014; Shergis et al., 2015). These approaches provide better coverage compared with molecular and chemical-based approaches, but their performance is low because they cannot directly consider complex molecular mechanisms and chemical structures. Moreover, the effects of reporting bias, sampling variance, and response variance should be considered to perform statistical analysis based on reporting data (DuMouchel, 1999; Bate and Evans, 2009; Tatonetti et al., 2012). Alternatively, machine learning-based approaches were proposed to utilize large volume of information. These approaches predicted the potential effects of natural compounds by investigating the drugs having similar properties to those of natural compounds (Rupp et al., 2010; Romano and Tatonetti, 2019; Chen and Kirchmair, 2020; Zhang et al., 2020). To construct prediction models, they applied classification algorithm, such as logistic regression, random forest, neural network, and support vector machine (SVM). However, limited natural compound information is still a bottleneck when trying to utilize various types of features in the learning process. In conclusion, we need to solve the problem with the bottleneck effect caused by the limited natural compound information and inappropriate methods available currently.

In this paper, we propose a deep learning-based approach to predict the medicinal uses of natural compounds. Our previous studies have shown that the various properties of natural compounds, such as molecular and chemical properties, can be utilized to predict the medicinal uses of natural compounds (Noh et al., 2018; Yoo et al., 2018a; Yoo et al., 2018b; Yoo et al., 2018c). Therefore, we adapted our previous approaches to extract the molecular and chemical properties of natural compounds (Supplementary Section S1 in Supplementary Data S1). Moreover, additional information was extracted by capturing latent knowledge from scientific literature to complement the incomplete molecular and chemical information. However, it is still difficult to perform integrated analysis because the extracted information is complex and heterogeneous. Also, the number of extracted features are relatively large comparing with the number of samples of training dataset. To solve this problem, we applied a partially connected deep neural network approach. The complex and heterogeneous information can be captured and analyzed by constructing multiple hidden layers in the deep learning model. For all approved and investigational drugs, we extracted latent knowledge, molecular interactions, and chemical property features and used them as inputs of the model. To predict the medicinal use of natural compounds, we used medicinal effects of drugs as the output class labels. Finally, the medicinal uses of 4,507 natural compounds for 15 diseases were predicted by the trained deep learning model. The evaluation results showed that a large number of predictions were successfully identified with high accuracy, sensitivity, and specificity. To conclude, the novelty of the present study is three-fold. Firstly, it is the first deep learning-based approach that identifies the medicinal uses of natural compounds. Secondly, it can be used to perform a large-scale natural compound study by utilizing large amounts of heterogeneous information, including latent knowledge, molecular interactions, and chemical properties, to mitigate the inadequacies of incomplete information, which causes a bottleneck effect. Finally, this approach can be used in a preliminary screening of natural compounds from a large number of candidates.

## Materials and Methods

### Data Collection

Plant-derived natural compounds and their chemical structure information were collected from KTKP (Portal, 2020), TCMID (Xue et al., 2012), COCONUT (Yoo et al., 2018a), and FooDB (FooDB, 2020). Drug information, including chemical structure and indication, was collected from DrugBank version 5.1.5 (Wishart et al., 2018). The molecular targets of the drugs and natural compounds were collected from the DrugBank, CTD (Davis et al., 2011), MATADOR (Günther et al., 2008), STITCH (Kuhn et al., 2013), and TTD (Zhu et al., 2011) databases. In this study, we used 4,507 natural compounds and 2,882 approved and investigational drugs that have at least five molecular target information. For extracting latent knowledge from scientific literature, we collected 13,200,786 PubMed abstracts that were published from 1950 to 2019, containing 236,645,741 sentences and 3,689,111,651 words. For the molecular interaction analysis, a protein-protein interaction (PPI) dataset was obtained from BioGrid version 3.5.182, containing 18,008 nodes and 504,848 edges (Chatr-Aryamontri et al., 2015).

### Generating Heterogeneous Features of Drugs and Natural Compounds

**FIGURE 1:**
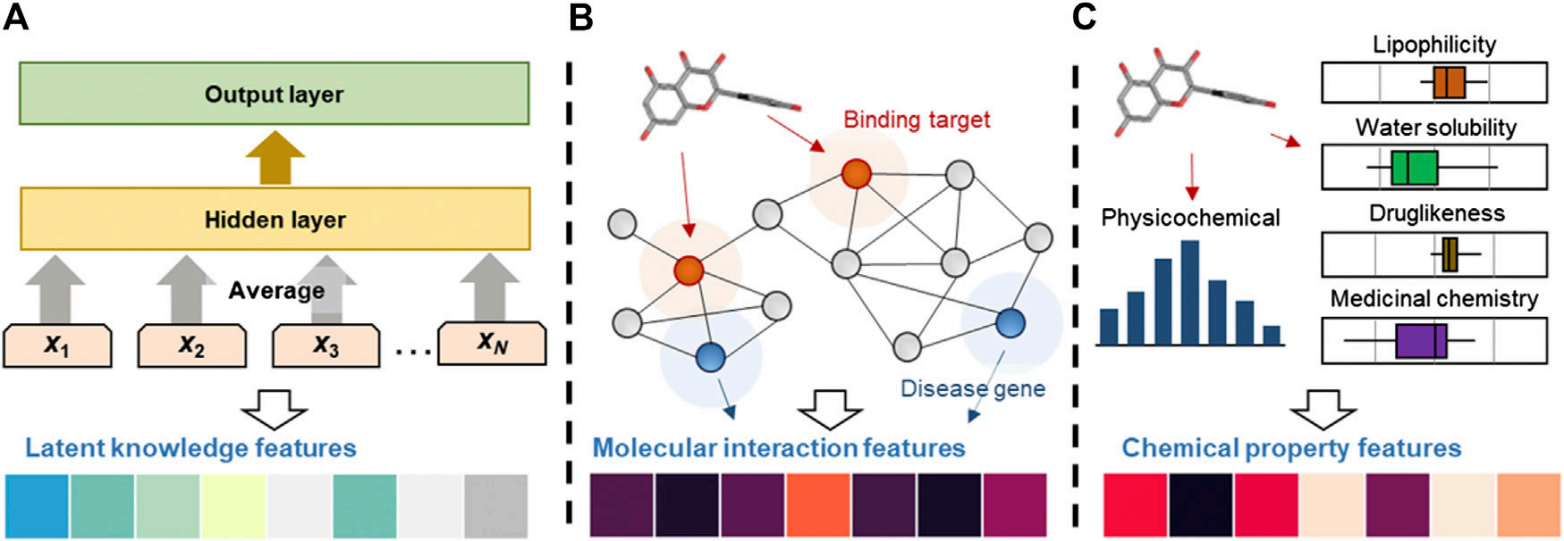
Computational processes for the generation of latent knowledge, molecular interaction and chemical property features. **(A)** Latent knowledge features were obtained by applying the text mining method to the PubMed abstracts. The n-gram features (x1, …, xN) are embedded and averaged to fore the hidden variable. **(B)** Molecular interaction features were generated by applying RWR algorithm to the PPI network. The RWR algorithm propagated compound effects from seed nodes (binding information, red circles) to their neighbors, recursively. **(C)** Chemical features, including physicochemical properties, lipophilicity, water solubility, pharmacokinetics, druglikeness and medicinal chemistry information, were calculated.

In this study, we generated three important features that can help us predict the medicinal effects of natural compounds (Figure 1). Each feature was generated by a fixed-length numeric vector form. We have provided the latent knowledge, molecular interaction, and chemical property features of the drugs and natural compounds in (https://doi.org/10.6084/m9.figshare.12671870).

#### Identification of Latent Knowledge Features by Text Mining

We generated latent knowledge features to obtain various types of drug and natural compound information from scientific literature. To this end, we applied a word embedding approach that represents a single word as a real-valued vector in a low-dimensional space (Figure 1A). There are several machine learning-based approaches for word embedding. For example, the word2vec creates embedding vectors of words in a given corpus using context to predict a word (continuous bag-of-words, C-BOW model) or using a word to predict the context (skip-gram model) (Mikolov et al., 2013a; Mikolov et al., 2013b). However, this method is highly dependent on the training corpus, making its application to rare or unusual natural compound and drug names difficult. In particular, the organic chemistry field includes many complex and compound words, such as “alpha-isothiocyanatotoluene.” Thus, the word2vec model cannot be used to appropriately estimate vector representations in the field. To solve this problem, we used fastText: a word representation using the sub-word skip-gram model that learns representations for character *n*-grams based on unlabeled corpora where each word is represented as the sum of the *n*-gram vector representations (Bojanowski et al., 2017; Young and Rusli, 2019). This model improves the representations of rare words by considering the character level information and internal structure of the words. For example, the natural compound name “alpha-isothiocyanatotoluene” can be estimated by dividing the word into “alpha,” “isothiocyanato,” and “toluene,” which are relatively frequent in the training corpora. The fastText model learns the distributed representations for all character *n*-grams in “alpha-isothiocyanatotoluene” and integrates the sub-word vectors to generate the final embedding vector of “alpha-isothiocyanatotoluene.” In this study, we used the pre-trained fastText model with Wikipedia and Common Crawl (Grave et al., 2018). The model additionally learned from the DrugBank indication and PubMed literature. Before training, we pre-processed the PubMed literature by tokenizing each word and transforming it into lowercase. We then transformed special characters and Greek symbols to alphabetic names (e.g., α to alpha) for generalization.

#### Identification of Molecular Interaction Features from Protein-Protein Interactions

We generated molecular interaction features by investigating mechanisms from the binding targets of compounds to the therapeutic targets or biomarkers of diseases. To this end, we constructed a PPI network and applied the random walk with restart (RWR) algorithm to quantify the molecular interaction effects of the compounds (Figure 1B). The RWR simulates the random walker starting from seed nodes and iteratively diffuses the node values to the neighbors according to edge weights until stability is achieved (Köhler et al., 2008; Li and Patra, 2010). The RWR is defined as the following equation.

$$p_{t+1}=\left(1-r\right)W^{T}p_{t}+rp_{0}$$

where W is the column-wise normalized adjacency matrix of the network, and r is the restarting probability of the random walker at each time step (it was set to 0.7 in this study). The adscript of pt represents the probability vector of each node at time step t, and p0 represents the initial probability vector. To apply the RWR algorithm, we first set the initial values of the seed nodes based on the binding target information of the compounds. This study used two types of binding target information: direct and indirect binding. Direct binding indicates the target proteins of the compounds, whereas indirect binding includes the molecular effects of the compounds, including changes in protein expression and compound-induced phosphorylation, or the effects of compounds that are transformed into active metabolites. By considering both types of binding information, we can consider the various properties of the compounds on the network. The initial values (p0) of direct and indirect binding were assigned as 1 and 0.3, respectively. Next, the transition probability from a node to the neighbors was calculated. We assumed that the transition probability represents the propagated effects on the PPI network. Based on Eq. 1, the transition probability vector of each node at time step t + 1 was calculated. The RWR algorithm simulated the random walker until pt became stable, which was evaluated by $p_{t+1}-p_{t}<10^{-8}$. In this study, we considered 4,487 disease-related proteins from a total of 18,008 proteins. Next, principal component analysis (PCA) was performed on the probability vector of proteins to reduce the dimensionality (i.e., from 4,487 to 285), as the number of proteins was still large compared with the number of instances of the training set (Jolliffe, 2003). In this study, we set the threshold of the cumulative explained variance ratio as 0.8. Finally, we generated molecular interaction features based on the PCA result.

#### Identification of Chemical Property Features Containing Physiological and Physicochemical Properties

Chemical property features were generated by considering physicochemical properties, lipophilicity, water solubility, pharmacokinetics, drug-likeness, and medicinal chemistry friendless information (Figure 1C). Physicochemical properties include molecular weight, number of heavy atoms, fraction Csp3, rotatable bonds, hydrogen-bond acceptors, hydrogen-bond donors, and molar refractivity. For all physicochemical properties, we performed feature scaling by applying Z-score normalization. The scale of input variables used to train the model is an important factor because unscaled inputs can result in a slow or unstable learning process, which causes exploding gradients in the learning process. Therefore, we performed Z-score normalization, which can standardize the values having zero-mean and unit variance. Lipophilicity contains the results of five different methods for the prediction of the partition coefficient between *n*-octanol and water (log P_o/w_), containing XLOGP3, WLOGP, MLOGP, SILICOS-IT, and iLOGP (Moriguchi et al., 1992; Moriguchi et al., 1994; Wildman and Crippen, 1999; Cheng et al., 2007; Sanders et al., 2012; Daina et al., 2017). The consensus log P_o/w_ is the arithmetic mean of the values predicted by the above five methods. Water solubility includes the results of three different methods for the prediction of water solubility, containing the ESOL, Ali, and SILICOS-IT methods (Delaney, 2004; Ali et al., 2012; Sanders et al., 2012). Pharmacokinetics includes human intestinal absorption, blood-brain barrier permeability, permeability glycoprotein (P-gp) substrate, five major isoforms of cytochrome P450 (i.e., CYP1A2, CYP2C19, CYP2C9, CYP2D6, and CYP3A4), and the logarithm of skin permeability coefficient (log *K*_p_). Drug-likeness contains Lipinski’s rule of five, Ghose, Veber, Egan, Muegge, and bioavailability score (Ghose et al., 1999; Egan et al., 2000; Muegge et al., 2001; Veber et al., 2002; Martin, 2005). We used lipophilicity, water solubility, pharmacokinetics, and drug-likeness values without feature scaling because the data are log scale or the data type was categorical. All categorical data were transformed into binary variables by applying one-hot encoding. Lastly, medicinal chemistry friendless contains the pan assay interference compounds (PAINS) filter (Baell and Holloway, 2010), the Brenk filter (Brenk et al., 2008), lead-likeness (Teague et al., 1999), and synthetic accessibility (Ertl and Schuffenhauer, 2009). All the properties were calculated using SwissADME (Daina et al., 2017).

#### Deep Learning-Based Prediction of the Medicinal Uses of Natural Compounds

**FIGURE 2:**
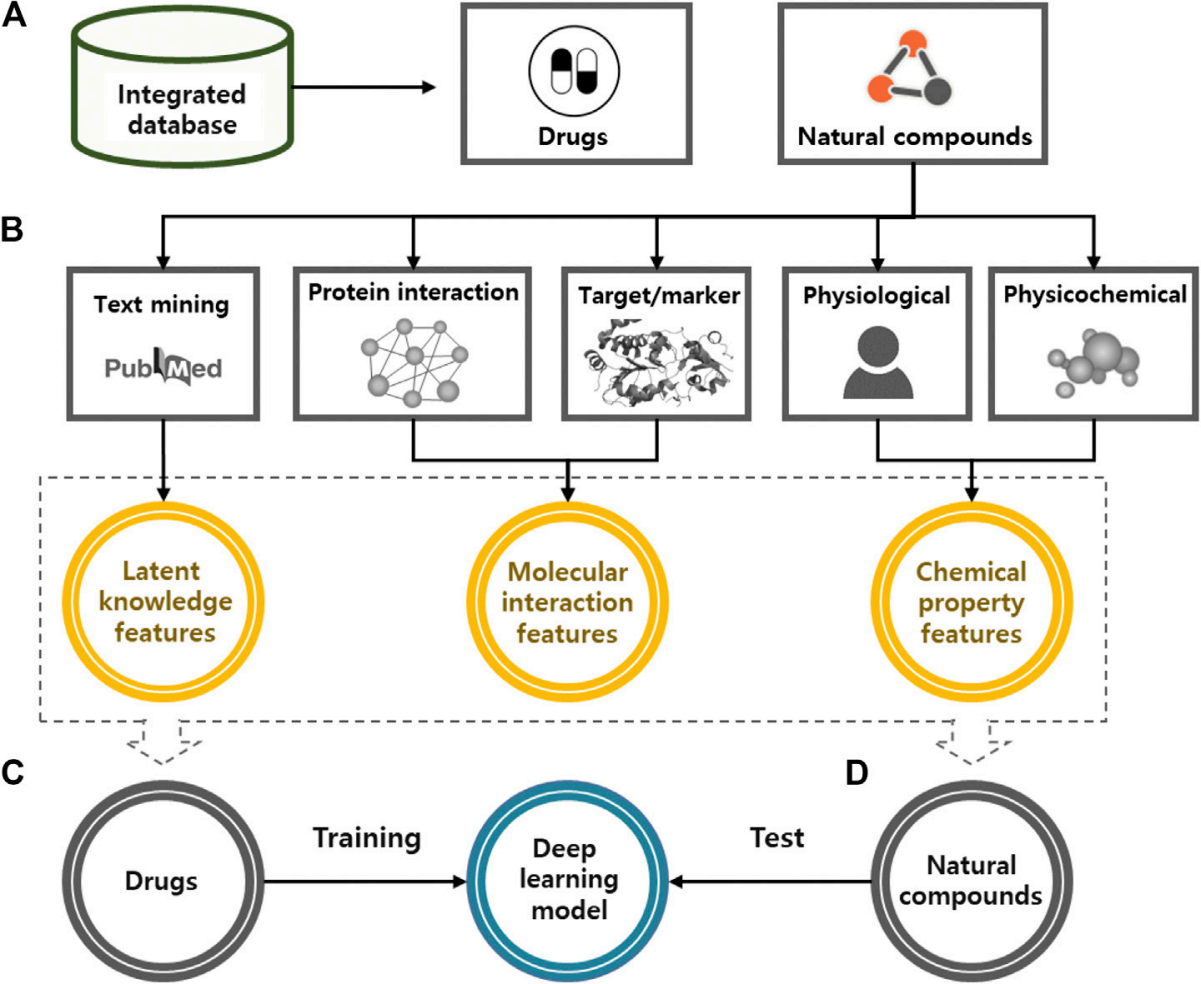
A systematic procedure of deep learning model for the identification of medicinal use of natural compounds. **(A)** We constructed an integrated database to merge various types of drug and natural compound information. **(B)** For all natural compounds and drugs, input features were generated based on the latent knowledge, molecular interaction and chemical property information. **(C)** We trained a deep learning model by using the extracted features and known efficacy of approved drugs. **(D)** Potential medicinal use of natural compounds was predicted by applying extracted features of natural compounds to the trained model.

In this study, we used a deep learning model to predict the potential medicinal effects of natural compounds (Figure 2). For all natural compounds and drugs, the algorithm works in four steps: 1) collecting various types of natural compound and drug information from public databases; 2) generating latent knowledge, molecular interaction, and chemical property features from the collected information via text mining, network analysis, and chemical property analysis; 3) training the deep learning model based on the features of the approved and investigational drugs as inputs and their indication information as outputs; and 4) predicting the medicinal uses of natural compounds based on the trained deep learning model.

**FIGURE 3:**
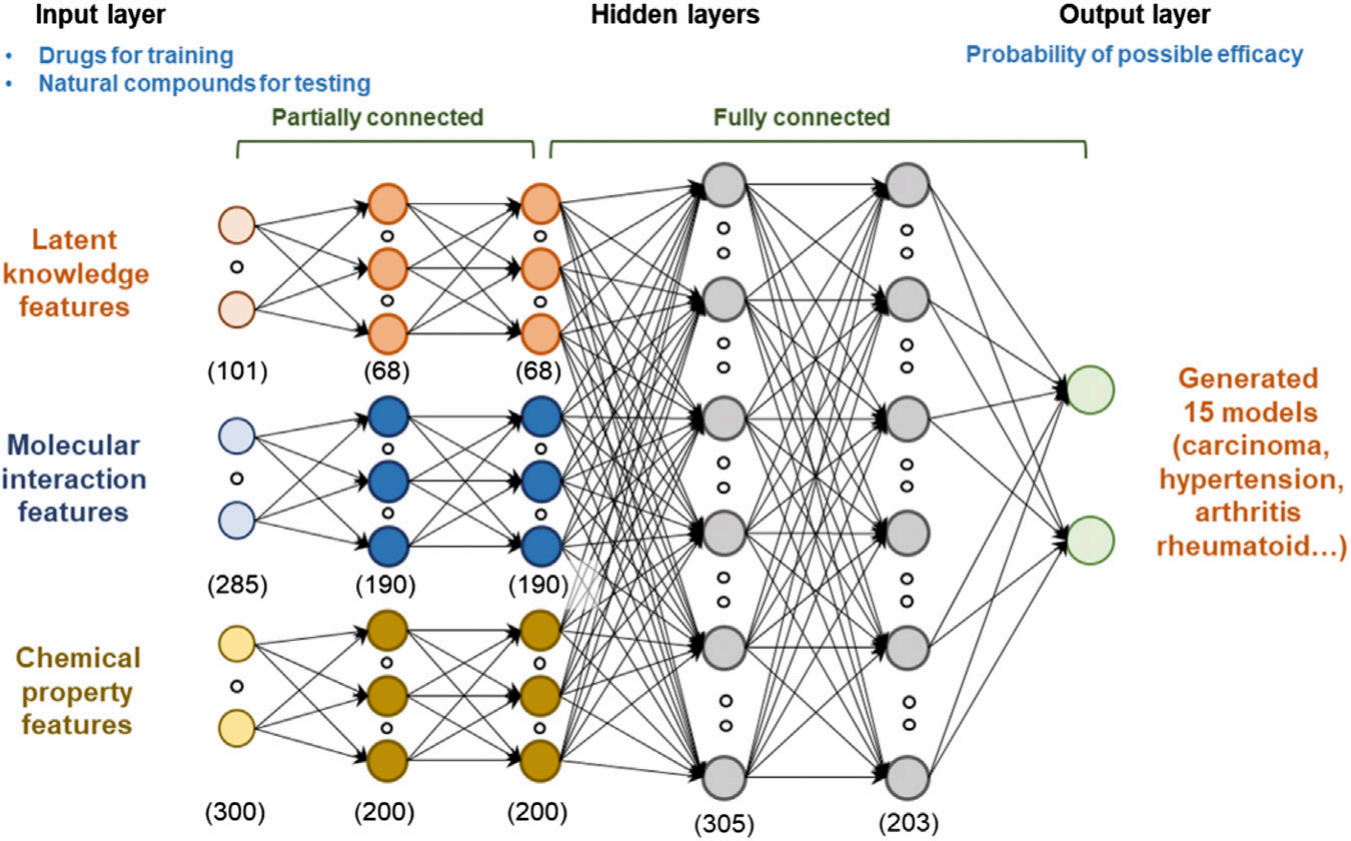
Architecture of the deep learning model for predicting the potential effects of natural compounds. We used latent knowledge, molecular interaction, and chemical property features as the inputs of the model, and each feature consisted of 101, 241, and 300 fixed‐length numeric vectors. To capture the high-level representation of each feature, we applied both partially connected and fully connected neural network structures. The model was trained based on the extracted features and verified indication information of drugs. Models were generated for 15 diseases, respectively. Finally, we predicted the potential medicinal effects of natural compounds based on the trained model.

When the input features are complex and heterogeneous, deep learning can improve the performance of the predictor by learning high-level representation from low-level features. The proposed model consists of four sequential layers (Figure 3): 1) input layer, 2) partially connected hidden layers, 3) fully connected hidden layers, and 4) output layer. The models were generated for 15 diseases, respectively, to predict the potential effects list from input features. For each drug or natural compound, we generated latent knowledge, molecular interaction, and chemical property features and used them as the inputs of the model. Hidden layers generalized their outputs by providing a high-level representation that was more abstract than the previous layer by discovering nonlinear relationships between the low- and high-level data. Let *X*_*l*_ is the output of the *l*th hidden layer. The forward propagation of the neural network with *l*th hidden layer can be represented as follow.

$$X_{l}=f\left(W_{l}X_{l-1}+b_{l}\right)$$

where *W*_*l*_ = [w_*l*1_, w_*l*2, … ,_ w_*l*n_] is the weight matrix of the edge from *l*-1st layer to *l*th layer, *b*_*l*_ is the bias of each hidden units, and *f* (·) is the activation function. In this study, the hidden layers were divided to two parts: the partially connected and fully connected parts. A fully connected neural network is the most commonly used model because it usually does not need a priori information on input data for defining the structure of the model (Shanmuganathan, 2016). This simplifies the model design since every neuron in one layer connecting to every neuron in the next layer. However, it may need large training data, and cannot consider the characteristic of the input feature types. A partially connected neural network can be defined as a network that contains only a subset of all possible connections. It has strengths in reducing complexity and improving generalization without producing significant modeling errors. This study applied a partially connected network to learn the spatially distinguished representation of each feature (Chen et al., 2016; Mason et al., 2018; Tek, 2018). When input neurons connect to the next layer of neurons, we set them to connect only neurons of the same input feature type. In the above-mentioned weight matrix (*W*_*l*_), zero values are set for the disconnected edges based on feature types. When *n* input features are fully connected to *m* neurons included in the hidden layer, *n*·*m* edges are created, but the proposed method creates $\underset{i}{\sum }n_{i}\cdot m_{i}$ edges (where *i* is the number of feature types). In this study, the partially connected model generated (101·68) + (285·160) + (300·200) edges, whereas the fully-connected model generated (101 + 285 + 300)·(68 + 190 + 200) edges. We applied a partially connected structure to the first and second hidden layers. This process reduced the number of edges to be trained by about 37%. Therefore, we can learn the weights of the edges with a relatively small training set taking into account the input feature types. The outputs of each partially connected layers are further concatenated to produce the single layer.

The proposed model was constructed using the following techniques. We applied the ReLU (Rectified Linear Unit) activation function in which *f*(*x*) = max (0, *x*) to all hidden units to increase the nonlinearity (Nair and Hinton, 2010). The weights were initialized using random numbers with zero-centered Gaussian with standard deviation of $\sqrt{2/n_{l}}$ (where *n*_*l*_ is the number of input units) that takes into account the ReLU nonlinearity (He et al., 2015). The batch normalization was used to normalize the input layer by re-centering and re-scaling (Ioffe and Szegedy, 2015). The class-weighted binary cross-entropy loss function for gradient descent was used to handle imbalanced dataset and defined as follow equation.

$$L_{w}=-\underset{i}{\sum }w_{0}y_{i}log\left(\hat{y_{i}}\right)+w_{1}\left(1-y_{i}\right)log\left(1-\hat{y_{i}}\right)$$

where *i* is the number of samples, $\hat{y_{i}}$ is the predicted model output, and $y_{i}$ is the corresponding target value. *w*_0_ and *w*_1_ are the weights for class 1 and 0, which are set to be inversely proportional to the class frequencies. To optimize the loss function, the Adam optimizer was applied with the learning rate = 0.0001, the learning rate decay = 0, *β*_1_ = 0.9 and *β*_2_ = 0.999 (Kingma and Ba, 2014). To avoid overfitting, early stopping was applied to an iterative procedure of gradient descent (Prechelt, 1998; Yao et al., 2007). We ran the models for 3,000 epochs and the batch size of 64 with early stopping (patience = 30).

We used a total of 2,882 approved and investigational drugs to train the model and 4,507 natural compounds for testing. To train the model, the output layer needed data indicating the effects of the drugs. As the indication information in DrugBank is described using free text, named entity recognition (NER) was applied to extract disease terms with standard identifiers. We used a Bidirectional Encoder Representations from Transformers (BERT)-based NER tool, known as BERN, to extract the disease terms from the drug indications (Kim et al., 2019; Lee et al., 2019). The extracted disease terms were mapped to Medical Subject Headings (MeSH) IDs and then converted into class labels (Lipscomb, 2000). For each drug, an average of 2.57 ± 0.11 (confidence interval = 0.95) MeSH IDs were mapped. All the NER results are provided in Supplementary Data S2. In this study, out of a total of 1,607 diseases, 15 disease terms that most frequently appeared in the indication information of drugs were used for predictions. We have provided the runnable source code in https://doi.org/10.6084/m9.figshare.13153184.

## Results

### Generated Latent Knowledge, Molecular Interaction, and Chemical Property Features

#### Latent Knowledge Features

We evaluated the latent knowledge features by calculating the similarity for groups of drugs based on the Anatomical Therapeutic Chemical (ATC) code. The ATC classification system categorizes drugs into different groups according to their chemical, pharmacological, and therapeutic properties (Methodology, 1982; Organization, 2019). In the ATC classification system, drugs are classified into groups at five different levels: the first level has 14 anatomical main groups; the second level indicates the main therapeutic group; the third level indicates a therapeutic or pharmacological subgroup; the fourth level indicates a therapeutic, pharmacological, or chemical subgroup; and the fifth level is the chemical substance. In this experiment, we grouped the drugs based on the five levels of the ATC code, respectively. For each group, cosine similarity values for the latent knowledge features of all possible drug pairs were calculated. From the result, we found that the mean value of the cosine similarity of the same ATC code group (*S*_1st_ = 0.417, *S*_2nd_ = 0.478, *S*_3rd_ = 0.551, *S*_4th_ = 0.603, *S*_5th_ = 0.608) was higher than that of the randomly selected group (*S*_random_ = 0.341–0.369). Moreover, it was confirmed that the similarity of the latent knowledge features increased as the level of ATC codes went from top to bottom. We have provided the results of cosine similarity for all groups in Supplementary Data S3. Moreover, our approach has a higher similarity values comparing with the word2vec method (*S*_1st_ = 0.322, *S*_2nd_ = 0.349, *S*_3rd_ = 0.423, *S*_4th_ = 0.498, *S*_5th_ = 0.502). These results indicated that the latent knowledge features effectively represented the anatomical, therapeutic, and pharmacological properties, as the deeper the ATC level, the more similar the properties of the drugs.

#### Molecular Interaction Features

We confirmed whether the molecular interaction features can be used to predict the potential medicinal effects of compounds. To this end, we mapped the sum of the protein values of the molecular interaction features to diseases based on the therapeutic target and biomarker information of diseases. Target diseases include 3,832 diseases defined by MeSH and Online Mendelian Inheritance in Man (OMIM) (Hamosh et al., 2005). Through this process, we obtained a list of disease scores for each drug. We then compared our predictions with the results of the network-based efficacy screening methods, including closest, shortest, kernel, center, and separation methods (Guney et al., 2016). The closest method predicts effects by calculating the mean shortest distance between compound targets and the nearest disease gene. The shortest method calculates the mean shortest distance between all compound targets and disease-related proteins. The kernel method calculates the distance by downweighting long paths exponentially. The center method calculates distance with considering the largest closeness centrality among the disease-related proteins. Lastly, the separation method calculates the sum of the mean distance between compound targets and disease-related proteins using the closest method and subtracts it from the mean shortest distance between compound targets and disease-related proteins. The results indicated that our predictions, which used the molecular interaction features, exhibited better performance (the area under the receiver operating characteristic, AUROC = 0.776 ± 0.094) than the closest (AUROC = 0.721 ± 0.076), shortest (AUROC = 0.697 ± 0.102), kernel (AUROC = 0.713 ± 0.084), center (AUROC = 0.707 ± 0.088), and separation (AUROC = 0.710 ± 0.078) in terms of medicinal effects prediction. These results indicated the effectiveness of the molecular interaction features in predicting the effects of compounds by analyzing propagated effects compared with the conventional approach.

#### Chemical Property Features

We performed various statistical tests to analyze the characteristics of the chemical property features. Firstly, we compared the distribution of the chemical properties of the natural compounds and drugs (Figure S1 in Supplementary Data S1). The results indicated that the median values of 68% chemical properties of natural compounds lie inside of the interquartile range of drugs. The mean, standard deviation, and standard error of the mean values of the chemical properties of the natural compounds and drugs are provided in Table S1 in Supplementary Data S1. Secondly, we compared the average similarity between compounds with the same medicinal effects and randomly selected drugs. It was confirmed that the average similarity of compounds with the same medicinal effect was 0.259 ± 0.031, whereas the average similarity of randomly selected compounds was 0.091 ± 0.014. This result indicated that the chemical properties of compounds with the same medicinal effect were likely to be similar.

### Performance Evaluation

Our method provided a list of the effects of the natural compounds with quantified scores. To assess the predictive performance, the AUROC and accuracy were calculated. We tested the performance for two different types of model structure and four different types of input data: 1) partially connected model using all features; 2) fully connected model using all features; 3) fully connected model using the latent knowledge feature only; 4) fully connected model using the molecular interaction feature only; 5) fully connected model using the chemical property feature only.

**FIGURE 4:**
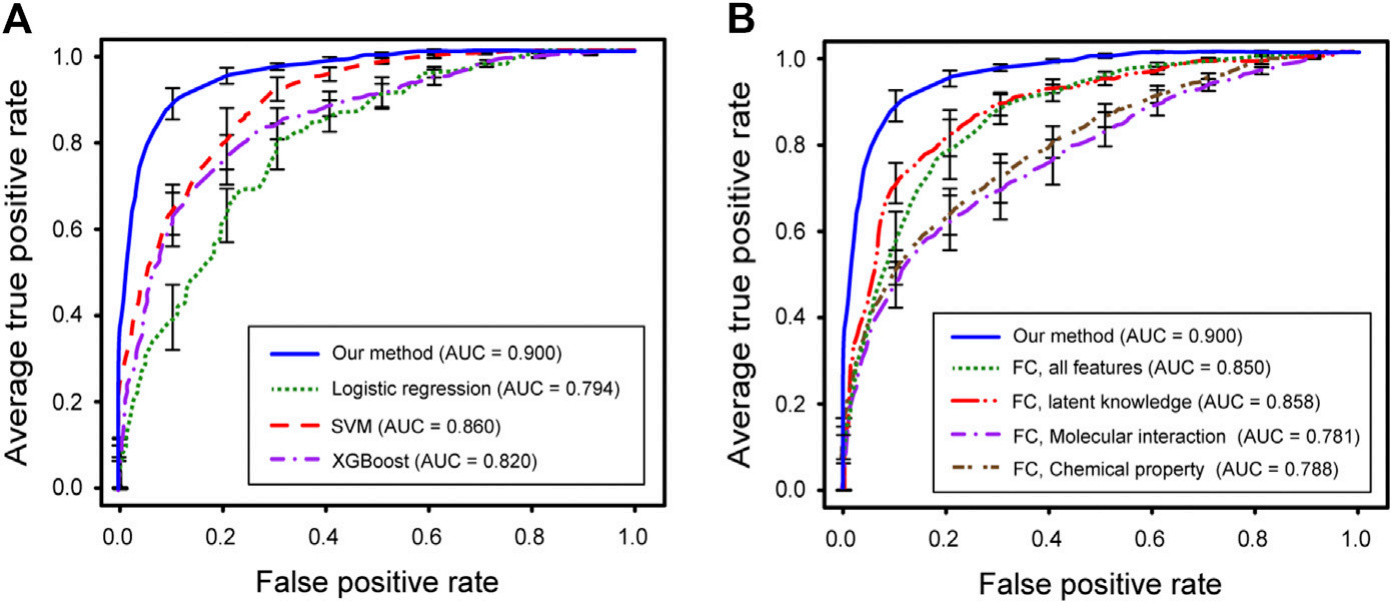
Performance evaluations of predicted medicinal effects of natural compounds. **(A)** ROC curves for our method (blue), logistic regression (green), SVM (red), and XGBoost (purple). **(B)** ROC curves for our method (blue), fully-connected using all feature (green), fully-connected only using lagent knowledge (red), fully-connected only using molecular interaction (purple), and fully‐connected only using chemical property (brown).

We first performed 10-fold cross-validation using only drug information. The drugs were divided in a ratio of 6:2:2 to train, validate, and test the model, respectively. As a result, AUROC values for 15 diseases were obtained (Table 1). Importantly, the partially connected model using all features (avg. AUROC = 0.900 ± 0.040) exhibited better performance than the method using only single information (avg. AUROC = 0.781 ± 0.077–0.858 ± 0.042) (Figure 4A). However, the fully connected model using all features (avg. AUROC = 0.850 ± 0.054) was worse performance than the fully connected model using the latent knowledge feature only. This is because the number of training samples is insufficient compared to the number of weights to be learned in fully connected model using all features. We further compared the method using the partially connected model with the fully connected model. The result indicated that the proposed partially connected model performed better than the fully connected model. This is because the partially connected neural network can be trained by a relatively smaller data set compared to a fully connected model. Lastly, we compared our method with other machine learning methods, including logistic regression, SVM, and bootstrapping (Table 2). Each model was created using all the features. The result showed that our method performed better than other machine learning methods (avg. AUROC = 0.781 ± 0.077–0.858 ± 0.042) (Figure 4B). Moreover, the average accuracy of the proposed model for 15 diseases was 0.971 ± 0.011. These results indicated that the proposed model was well built by reflecting the characteristics of the heterogeneous information. Next, we confirmed whether the model could be used to predict the medicinal effect of natural compounds (Table 3). We trained the model based on drug information and tested it using the verified medicinal effect information of natural compounds. Furthermore, an additional experiment was conducted using the inferred effects of the natural compounds as a test set because the verified medicinal effect information of natural compounds was limited. We found that the proposed deep learning model, which was trained using drug information, successfully predicted the verified (avg. AUROC = 0.832 ± 0.032) and inferred medicinal effects (avg. AUROC = 0.883 ± 0.033) of natural compounds. All predicted results, including a list of the effects of natural compounds with scores, are provided in Supplementary Data S4.

**TABLE 1:** AUROC values of the five different cases of the trained models in predicting the medicinal uses of drugs for 15 diseases.

Disease term	Partially connected	Fully connected			
	All features	All features	Latent knowledge features only	Molecular interaction features only	Chemical property features only
Carcinoma	0.774	0.684	0.767	0.702	0.711
Hypertension	0.970	0.962	0.955	0.882	0.777
Pain	0.943	0.776	0.840	0.815	0.611
Diabetes mellitus, type 2	0.850	0.765	0.824	0.564	0.616
Arthritis, rheumatoid	0.774	0.692	0.692	0.683	0.667
Urinary tract infections	0.985	0.983	0.948	0.986	0.944
Alzheimer’s disease	0.864	0.757	0.859	0.588	0.810
Bacterial infections	0.948	0.926	0.880	0.717	0.865
Parkinson’s disease	0.995	0.947	0.977	0.913	0.953
Heart failure	0.880	0.873	0.865	0.727	0.833
Sleep initiation and maintenance disorders	0.875	0.846	0.865	0.669	0.870
Skin diseases	0.774	0.789	0.759	0.587	0.653
Nausea	0.934	0.971	0.865	0.957	0.798
Myocardial infarction	0.964	0.798	0.800	0.975	0.766
Stroke	0.972	0.974	0.971	0.946	0.949
Average	0.900 ± 0.040	0.850 ± 0.054	0.858 ± 0.042	0.781 ± 0.077	0.788 ± 0.059

**TABLE 2:** Comparison of AUROC values of the proposed method with three machine learning-based methods, including logistic regression, SVM, and XGBoost.

Disease term	Proposed method	Logistic regression	SVM	XGBoost
Carcinoma	0.774	0.673	0.715	0.752
Hypertension	0.970	0.827	0.846	0.878
Pain	0.943	0.761	0.793	0.822
Diabetes mellitus, type 2	0.850	0.714	0.766	0.810
Arthritis, rheumatoid	0.774	0.653	0.688	0.725
Urinary tract infections	0.985	0.903	0.934	0.952
Alzheimer’s disease	0.864	0.772	0.817	0.831
Bacterial infections	0.948	0.851	0.826	0.916
Parkinson’s disease	0.995	0.910	0.952	0.963
Heart failure	0.880	0.813	0.807	0.833
Sleep initiation and maintenance disorders	0.875	0.751	0.796	0.855
Skin diseases	0.774	0.725	0.740	0.781
Nausea	0.934	0.812	0.912	0.892
Myocardial infarction	0.964	0.836	0.881	0.893
Stroke	0.972	0.915	0.964	0.967
Average	0.900 ± 0.040	0.794 ± 0.042	0.829 ± 0.043	0.858 ± 0.038

**TABLE 3:** AUROC values of the trained models in predicting the medicinal uses of natural compounds for 15 diseases using two different test sets.

Disease term	Verified effect	Verified and inferred effect
Carcinoma	0.767	0.813
Hypertension	0.912	0.935
Pain	0.871	0.903
Diabetes mellitus, type 2	0.793	0.822
Arthritis, rheumatoid	0.725	0.761
Urinary tract infections	0.846	0.910
Alzheimer’s disease	0.827	0.841
Bacterial infections	0.879	0.927
Parkinson’s disease	0.924	0.961
Heart failure	0.808	0.894
Sleep initiation and maintenance disorders	0.797	0.867
Skin diseases	0.718	0.785
Nausea	0.844	0.913
Myocardial infarction	0.902	0.947
Stroke	0.870	0.969
Average	0.832 ± 0.032	0.883 ± 0.033

We additionally performed the statistical analysis based on literature reporting the predicted medicinal effects of natural compounds (Table 4). We made three independent sets by selecting top-ranked 10%, bottom-ranked 10%, and randomly selected prediction results. Then, we confirmed whether the high-scored predictions have more evidence than the low-scored and randomly selected predictions. To do this, co-occurrences (*n*_c_) of natural compound and disease terms in PubMed abstracts were counted. The average co-occurrence frequency of the high-scored set (*n*_c_ = 0.87 ± 0.18) was 9.6 and 3.8 times larger than the low-scored set (*n*_c_ = 0.09 ± 0.03) and random set (*n*_c_ = 0.23 ± 0.11). Next, the co-occurrence was normalized as the Jaccard index (*JI*) by dividing the frequency of co-occurrence by the frequency of the union of individual terms to reduce the size influence associated with the term frequency (Eck and Waltman, 2009). The average Jaccard index of the high-scored set (*JI* = 1.07 × 10^−4^) was higher than those of the low-scored (*JI* = 2.17 × 10^−8^) and random set (4.31 × 10^−5^). Furthermore, we performed Fisher’s exact test to examine the significance of the predictions. Fisher’s exact test assess the null hypothesis (e.g., there is no difference in the proportions of predictions between natural compound and disease) of independence based on the hypergeometric distribution of the numbers in a contingency table (Agresti, 1992). To obtain the contingency table of each prediction, the number of PubMed abstracts was counted based on whether they included the natural compound and whether they included the target disease. The number of significant predictions of the high-scored set (*n*_*f*_ = 58.53 ± 14.01) was markedly larger than those of the low-scored (*n*_*f*_ = 13.46 ± 7.42) and random sets (*n*_*f*_ = 27.86 ± 9.98). Lastly, we performed the Mann-Whitney *U* test to confirm the statistical difference of above analysis among the high-scored, low-scored, and random sets was significant. A *p*-value of Mann-Whitney *U* test lower than 0.05 was considered statistically significant. The result indicated that all statistical analysis results were significantly different among the high-scored, low-scored, and random sets.

**TABLE 4:** The statistical analysis was performed by comparing co-occurrence, Jaccard index and Fisher’s exact test values among high-score, low-scored, and randomly selected sets. Statistical significance was calculated by the *p*-value of Mann-Whitney *U* test.

		Co-occurrence	Jaccard index	Fisher’s exact test ^a^
High-scored set		0.87 ± 0.18	1.07 × 10^−4^	58.53 ± 14.01
Low-scored set		0.09 ± 0.03	2.17 × 10^−8^	13.46 ± 7.42
Randomly selected set		0.23 ± 0.11	4.31 × 10^−5^	27.86 ± 9.98
Mann-Whitney *U* test (*p*-value)	H vs. L	<0.001	<0.001	<0.001
	H vs. R	<0.001	<0.001	<0.001
	L vs. R	<0.001	<0.001	<0.001

a *p*-value threshold of Fisher’s exact test is 0.001.

### Animal and Clinical Studies

In this study, the medicinal uses of natural compounds were identified by deep learning. To evaluate the predicted effects of the natural compounds, we performed evidence-based analysis (Table 5). Firstly, we investigated *in vitro* and animal studies. 5-Caffeoylquinic acid may prevent cognitive impairment in mice with Alzheimer’s disease (Ishida et al., 2020). Tangeretin may have therapeutic effects on rheumatoid arthritis in a rat model (Li et al., 2019). Gossypol family members, such as BH3 mimetics, may have benefits in the management of rheumatoid arthritis (Billard, 2013). Indolyl-methyl-glucosinolate was reported to exert anti-inflammatory activity (Vo et al., 2014), and gentianine showed low anti-inflammatory activity in carrageenan-induced hind-paw edema (Perez, 2001). Gambogic acid may ameliorate angiogenesis in mice with diabetic retinopathy (Cui et al., 2018). Gamma-oryzanol was shown to be safe and effective in improving the conditions of diabetes mellitus in several animal studies (Szcześniak et al., 2016). Octopamine may be involved in central blood pressure regulation (Delbarre et al., 1982). According to the reperfusion duration, route of administration, and timing of the pretreatment regimen, resveratrol showed benefits in the treatment of myocardial infarct-sparing (Mao et al., 2019). N-methyl-(R) salsolinol, as an endogenous neurotoxin, may induce Parkinson’s disease in rats (Naoi et al., 1997). The proliferation of MDA-MB-231 cells was prohibited using neohesperidin in a time- and dose-dependent manner in human breast adenocarcinoma (Xu et al., 2012). Tritiated norephedrine may inhibit the substitution of beta-phenylethylamines in rats (Henderson et al., 1995). Agmatine protected brain tissues from edema after cerebral ischemia in mice (Kim et al., 2010).

**TABLE 5:** Predicted pharmacological effects of natural compounds in each phenotype.

Disease	Compound	Animal and clinical studies
Alzheimer’s disease	4,5-dicaffeoylquinic acid	PMID: 32075202
	3,4-dicaffeoylquinic acid	PMID: 32075202
Rheumatoid arthritis	Tangeretin	PMID: 31344704
	Gossypol	PMID: 23974697
Bacterial infection	Indolylmethylglucosinolate	PMID: 24360830
	Gentianamine	PMID: 12805773
Carcinoma	Melatonin	PMID: 28415828
Diabetes mellitus, type 2	Gambogic acid	PMID: 29129773
	Gamma-oryzanol	PMID: 26718022
Heart failure	Ergosterol	PMID: 19753490
	Arginine	PMID: 15226784
Hypertension	Reserpine	PMID: 27997978
	Norepinephrine	PMID: 29915014
	Octopamine	PMID: 6125331
	Digitoxin	PMID: 26321114
Myocardial infarction	Resveratrol	PMID: 31182995
Nausea	Pyridoxine	PMID: 25884778
	Camphene	PMID: 29614764
Pain	Morphine	PMID: 8544547
	Carvacrol	PMID: 23791894
	L-menthol	PMID: 20171409
Parkinson’s disease	Salsolinol	PMID: 9120428
	dl-laudanosine	PMID: 8769881
Skin disease	Neohesperidin	PMID: 23285810
Sleep initiation and maintenance disorders	Norephedrine	PMID: 26321114
	Melatonin	PMID: 23691095
	Colchine	PMID: 14744269
Stroke	Aspirin	PMID: 31867054
	Agmatine	PMID: 20029450
Urinary tract infection	5-Methylcytosine	PMID: 7767983
	Cytosine	PMID: 2041144

Next, we checked clinical studies. Melatonin may enhance the therapeutic effects of various anticancer drugs (Li et al., 2017). Ergosterol biosynthesis inhibitors may have curative activities in murine models of acute and chronic Chagas disease (Urbina, 2009). In patients with chronic stable congestive heart failure, l-arginine prolongs the exercise duration (Bednarz et al., 2004). Reserpine may reduce systolic blood pressure as a first-line antihypertensive drug, as shown in a Cochrane review (Shamon and Perez, 2016). Plasma norepinephrine is directly related to muscle sympathetic nerve activity values in hypertensive group (Grassi et al., 2018). In a blind placebo-controlled trial, a pyridoxine-doxylamine combination appears to be safe for pregnant women suffering from nausea and vomiting associated with pregnancy (Koren et al., 2015). RCTs showed that *Zingiber officinale* Roscoe, which contains camphene, can be used to alleviate nausea and vomiting in pregnant women with no common side effects (Stanisiere et al., 2018). In a randomized double-blind crossover study, the use of oral morphine for pain control led to a reduction in pain intensity relative to placebo use (Moulin et al., 1996). Eugenol and carvacrol were shown to induce oral irritation, causing various types of pain (Klein et al., 2013). A single patch containing methyl salicylate and l-menthol significantly relieved the pain associated with mild to moderate muscle strain (Higashi et al., 2010). Laudanosine prevented NADH-linked mitochondrial respiration and complex I activity as a neurotoxin that promotes Parkinson’s disease (Morikawa et al., 1996). Melatonin decreases sleep onset latency, increases total sleep time, and improves overall sleep quality, as shown in a meta-analysis (Ferracioli-Oda et al., 2013). One case study revealed that long-term colchicine therapy leads to symptomatic respiratory muscle weakness (Tanios et al., 2004). Clopidogrel monotherapy leads to lower risks of major adverse cardiovascular or cerebrovascular events compared with aspirin treatment (Paciaroni et al., 2019). Demethylation of 5-Methylcytosine may help in the management of interstitial cystitis (Shahid et al., 2018). Flucytosine may serve as an effective and safe treatment for urinary tract infection (Fujihiro et al., 1991).

## Discussion

In recent years, natural compounds have received considerable attention as an important resource for the development of drugs and dietary supplements owing to the increasing evidence of their health-promoting effects. Therefore, numerous attempts have been made to determine the medicinal properties of natural compounds through scientific analysis. Most previous studies have focused on *in vitro* and *in vivo* approaches, but these approaches have limitations in terms of cost and time. As an alternative, *in silico* analysis has been proposed, but another bottleneck effect may occur owing to the heterogeneous and incomplete nature of the information on natural compounds.

Our previous studies have shown that natural compounds have relatively limited chemical and molecular information compared with drugs (Noh et al., 2018; Yoo et al., 2018a; Yoo et al., 2018b; Yoo et al., 2018c). Analyzing this incomplete information using conventional statistical methods can distort the results or limit the coverage. In addition, the combination of various types of information is difficult to consider. Thus, we applied the partially connected deep neural network to solve these problems. Our underlying hypothesis consisted of two parts. First, even if a certain type of information is incomplete, its effect can be mitigated by utilizing many other types of information in the learning process. In general, we believe that the more kinds of information we use, the better we can make the model. But it becomes difficult to consider the heterogeneous characteristics of the information. In addition, as the number of features increases, the number of samples required for learning increases. In other words, using a large number of features does not always improve the performance of the model. The prerequisite for this is that there must be a sufficient amount of samples compared to the number of features. As shown in the results of this study, when a fully connected neural network was trained using complex and heterogeneous features, the performance was rather poor than when fewer features were used. Therefore, this study applied partially connected structure to alleviate the incompleteness of natural compound information by applying heterogeneous and complex characteristics. This approach is meaningful in that it provides directions on how to utilize heterogeneous and complex information on natural compounds in the future study. Second, if a natural compound has similar properties to certain approved drugs, this compound is more likely to have medicinal effects similar to that of the drugs. According to the validation results, the model incorporating various types of information outperformed the models incorporating a single type of information. This indicated that the simultaneous processing of various types of information led to synergy in the deep learning model. If our approach did not mitigate the incompleteness of the information, the performance would have converged to the average of the models using a single type of information. Moreover, it was confirmed that the model trained with drug information can successfully predict the medicinal effects of natural compounds. These results supported our underlying hypothesis.

Our study had additional strengths in the following aspects. First, various types of natural compound and drug information, including latent knowledge, molecular interactions, and chemical properties, can be utilized in many other *in silico* studies. All of the information was not extracted under specific conditions or constraints; thus, they can be easily used in various fields. We expect that the information will help address the lack of information that natural compound-related studies have been experiencing. Moreover, it can be utilized in drug-related studies such as drug repositioning, drug-drug interactions, and drug-target identification. Second, we can perform bidirectional analysis, including both bottom-up and top-down analyses. Our approach was basically a bottom-up analysis, as it was possible to find medicinal natural compound candidates for disease treatment based on the model trained using the extracted natural compound information. Additionally, we can perform top-down analysis of the predicted results by investigating detailed characteristics, including molecular mechanisms, oral bioavailability, drug availability, and tissue specificity, based on the input features. In conclusion, our study provided a combination of top-down and bottom-up analyses for more precise prediction.

There are additional considerations that may improve our method. First, there was a limited number of drugs and natural compounds that were used as training and test sets in the deep learning model. In the training step, a total of 2,882 approved and investigational drugs were used, which is relatively small compared with the number of input features. To compensate for this problem, inferred compound-disease associations from the CTD database were used in training, but another problem still remained: the inferred information was relatively unreliable. Furthermore, in the test step, only 4,507 natural compounds were considered owing to the limited current knowledge on natural compounds. However, these problems will be solved as knowledge on natural compounds will accumulate in future experiments. Second, it was difficult to clearly interpret the exact manner in which the current deep learning model made predictive results. This problem has been raised continuously in the field of machine learning, and efforts have recently been made to solve it through layer-wise analysis (Montavon et al., 2010; Samek et al., 2017; Montavon et al., 2018). Therefore, we plan to apply the layer-wise analysis algorithm to the proposed model to interpret the predictions. With further improvements, we expect that our model will make more reliable predictions of the medicinal uses of natural compounds.

## Data Availability Statement

The original contributions presented in the study are included in the article/Supplementary Material, further inquiries can be directed to the corresponding author.

## Author Contributions

Conceptualization, SY, HY, and DL; methodology, SY and DL; software, SY, SL, JS, SM, and EL; validation, SY, HY, and SL; formal analysis, SY, HY, MS, and DL; investigation, SY, HY, and MS; resources, SY and DL; data curation, SY, SL, JS, and EL; writing original draft preparation, SY, MS, and DL; writing review and editing, SY, MS, and DL; visualization, SY and HY; supervision, MS and DL; project administration, DL; funding acquisition, DL. All authors have read and agreed to the published version of the manuscript.

## Funding

This research was supported by the Bio‐Synergy Research Project (NRF‐2012M3A9C4048758) of the Ministry of Science, ICT, and Future Planning, through the National Research Foundation, and supported by the National Research Foundation of Korea grant funded by the Korea government. (MSIT) (NRF‐2020R1C1C1006007). The authors declare no competing financial interests.

## Conflict of Interest

The authors declare that the research was conducted in the absence of any commercial or financial relationships that could be construed as a potential conflict of interest.
