# Supplementary material for: A Deep Learning-Based Approach for Identifying the Medicinal Uses of Plant-Derived Natural Compounds
Source: Front Pharmacol. 2020 Nov 30;11:584875. doi: 10.3389/fphar.2020.584875 (PMC7845697; doi:10.3389/fphar.2020.584875)
Supplement: Supplementary file 1 [file datasheet1.docx]

Supplementary Material

# Supplementary Section

## Characteristics of *in silico* natural compound study

In our previous studies, we have shown the following three characteristics of natural compounds (Noh et al., 2018;Yoo et al., 2018a;Yoo et al., 2018b;Yoo et al., 2018c). First, the potential effects of natural compounds can be predicted by analyzing the functional, molecular, or phenotypic properties. For example, we can identify the potential effects of natural compounds by applying network analysis to the molecular network. Furthermore, bioactive natural compound candidates can be found by determining the physicochemical and physiological properties. Second, when natural compounds have properties similar to those of certain drugs, these natural compounds have medicinal effects similar to that of the drugs. However, although this approach can estimate the potential effects of natural compounds, it has limited coverage because a large amount of natural compound information, such as molecular targets or chemical structure, remains hidden. Third, the bottleneck effect in natural compound studies, which results from the incompleteness of natural compound information, can be solved by efficiently utilizing heterogeneous natural compound information. To incorporate all the above mentioned considerations, we proposed a deep learning-based approach. This approach is meaningful because it can help alleviate the incompleteness of information by utilizing the heterogeneous information of natural compounds and drugs.

# Supplementary Figures and Tables

## Supplementary Figures

##
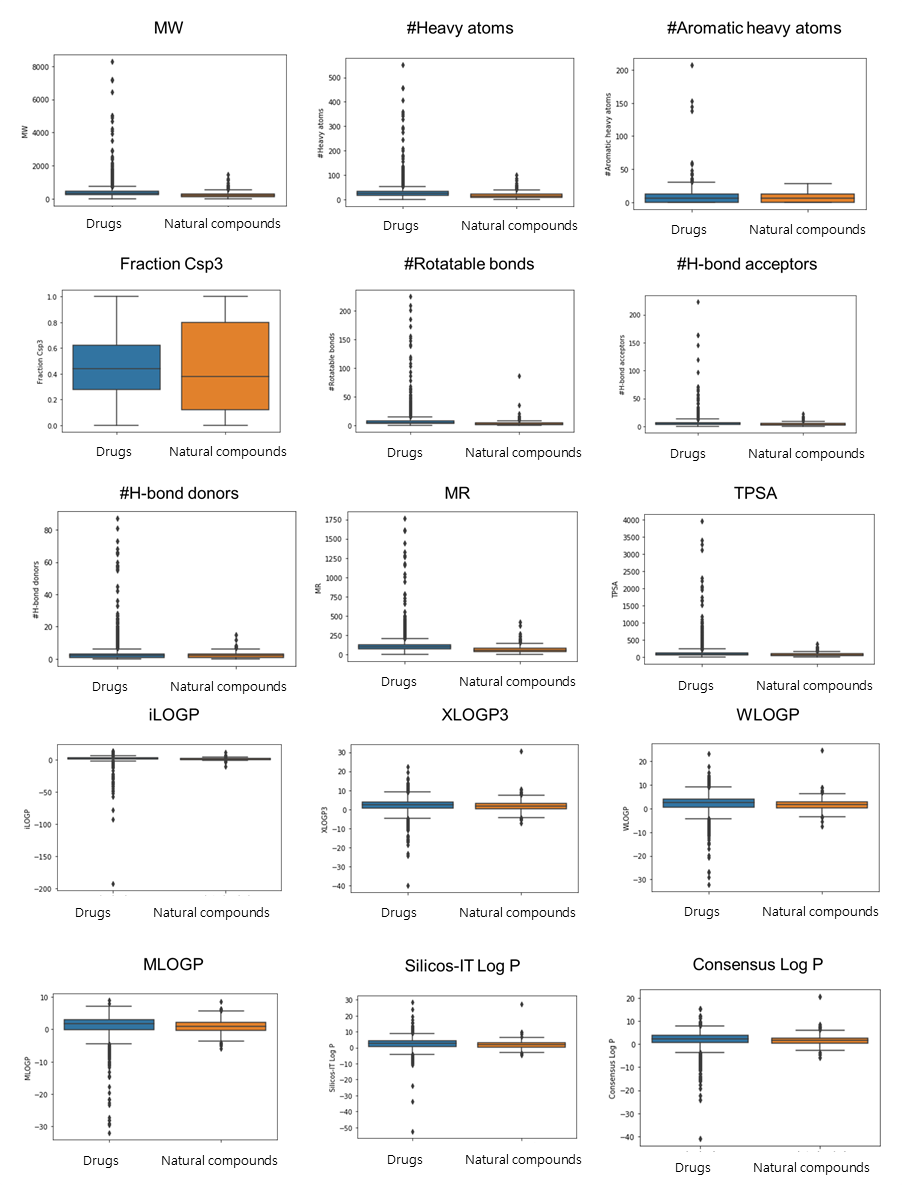


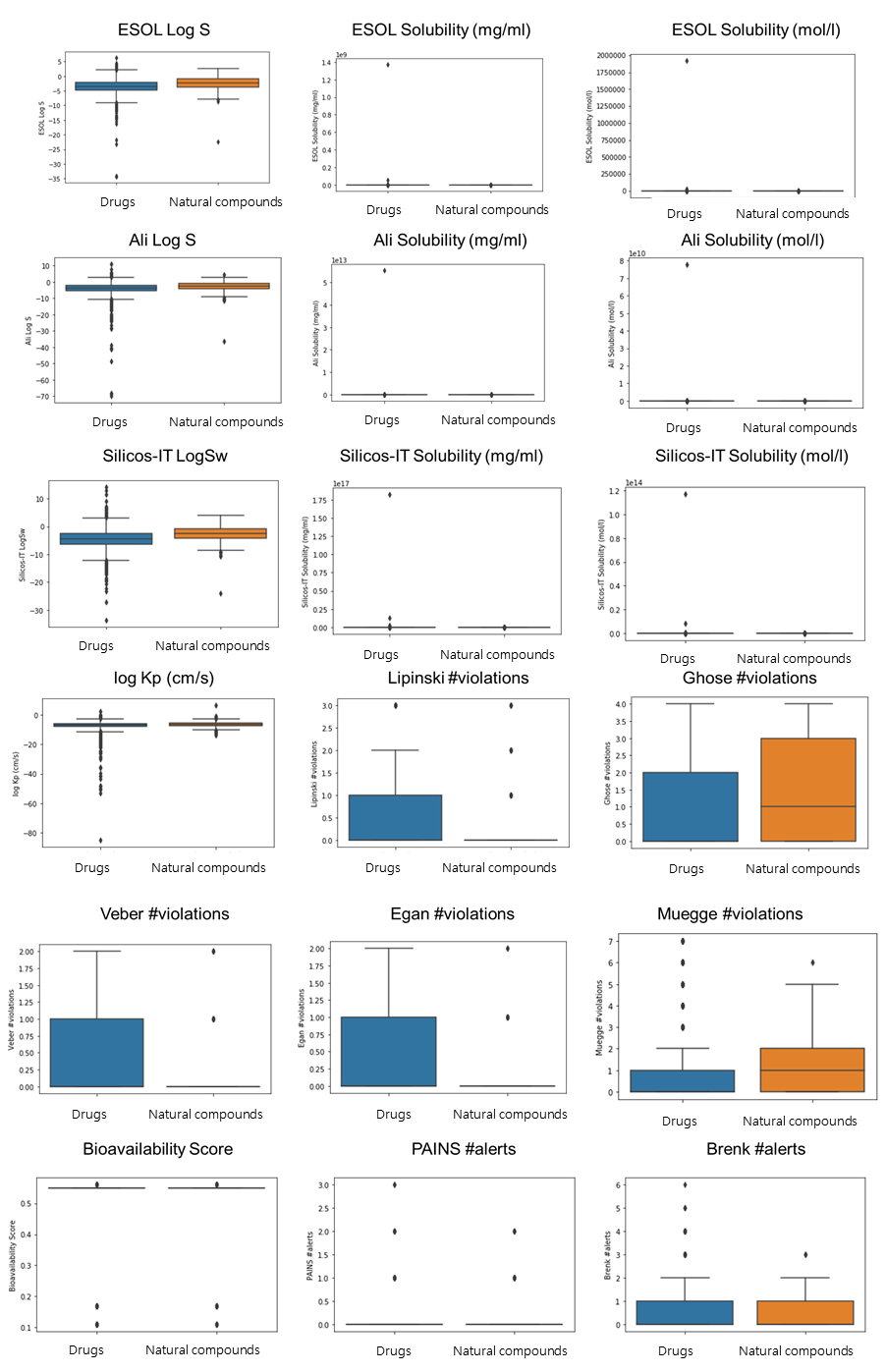


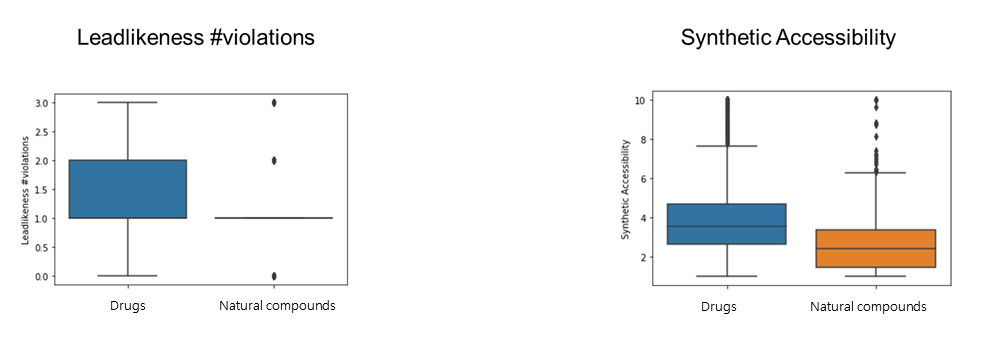


**Supplementary Figure 1.** The distribution of the chemical properties of the natural compounds (orange box plot) and drugs (blue box plot). We considered chemical properties including physicochemical properties, lipophilicity, water solubility, pharmacokinetics, drug-likeness, and medicinal chemistry information, accumulating up to 35 properties.

## Supplementary Tables

**Supplementary Table 1.** The mean, standard deviation, and standard error of the mean values of the chemical properties of the natural compounds and drugs.

| **Variables** | **Natural compounds** | | | **DrugBank** | | |
| --- | --- | --- | --- | --- | --- | --- |
|  | **Mean** | **Standard deviation** | **standard error of the mean** | **Mean** | **Standard deviation** | **standard error of the mean** |
| MW | 236.37 | 156.66 | 6.90 | 417.55 | 424.61 | 7.91 |
| #Heavy atoms | 16.77 | 11.09 | 0.49 | 28.44 | 28.79 | 0.54 |
| #Aromatic heavy atoms | 5.87 | 6.07 | 0.27 | 8.97 | 9.37 | 0.17 |
| Fraction Csp3 | 0.44 | 0.35 | 0.02 | 0.46 | 0.25 | 0.00 |
| #Rotatable bonds | 3.18 | 5.09 | 0.22 | 7.44 | 13.05 | 0.24 |
| #H-bond acceptors | 3.73 | 3.03 | 0.13 | 6.13 | 8.98 | 0.17 |
| #H-bond donors | 2.02 | 1.95 | 0.09 | 2.71 | 5.45 | 0.10 |
| MR | 64.68 | 42.64 | 1.88 | 109.77 | 105.10 | 1.96 |
| TPSA | 68.93 | 51.81 | 2.28 | 114.60 | 192.21 | 3.58 |
| iLOGP | 1.78 | 1.46 | 0.06 | 1.79 | 6.20 | 0.12 |
| XLOGP3 | 1.66 | 2.86 | 0.13 | 2.06 | 3.44 | 0.06 |
| WLOGP | 1.66 | 2.35 | 0.10 | 2.13 | 3.49 | 0.07 |
| MLOGP | 0.95 | 2.13 | 0.09 | 1.13 | 3.17 | 0.06 |
| Silicos-IT Log P | 1.80 | 2.32 | 0.10 | 2.46 | 3.15 | 0.06 |
| Consensus Log P | 1.57 | 2.09 | 0.09 | 1.91 | 3.02 | 0.06 |
| ESOL Log S | -2.39 | 2.24 | 0.10 | -3.52 | 2.70 | 0.05 |
| ESOL Solubility (mg/ml) | 1054.94 | 9228.83 | 406.67 | 507237.11 | 25549898.10 | 476094.24 |
| ESOL Solubility (mol/l) | 4.66 | 29.97 | 1.32 | 697.65 | 35781.71 | 666.75 |
| Ali Log S | -2.72 | 2.92 | 0.13 | -4.13 | 4.09 | 0.08 |
| Ali Solubility (mg/ml) | 8310.29 | 147912.99 | 6517.83 | 1.92E+10 | 1.03E+12 | 1.92E+10 |
| Ali Solubility (mol/l) | 54.41 | 1006.54 | 44.35 | 2.70E+5 | 1.45E+7 | 2.69E+5 |
| Silicos-IT LogSw | -2.57 | 2.55 | 0.11 | -4.53 | 3.48 | 0.06 |
| Silicos-IT Solubility (mg/ml) | 13462.50 | 181670.56 | 8005.36 | 6.85E+13 | 3.40E+15 | 6.34E+13 |
| Silicos-IT Solubility (mol/l) | 38.70 | 442.96 | 19.52 | 4.36E+10 | 2.19E+12 | 4.07E+10 |
| log Kp (cm/s) | -6.57 | 1.86 | 0.08 | -7.43 | 3.86 | 0.07 |
| Synthetic Accessibility | 2.71 | 1.66 | 0.07 | 3.93 | 1.92 | 0.04 |

# References

Noh, K., Yoo, S., and Lee, D. (2018). A systematic approach to identify therapeutic effects of natural products based on human metabolite information. *BMC bioinformatics* 19**,** 205.

Yoo, S., Ha, S., Shin, M., Noh, K., Nam, H., and Lee, D. (2018a). A Data-Driven Approach for Identifying Medicinal Combinations of Natural Products. *IEEE Access* 6**,** 58106-58118.

Yoo, S., Kim, K., Nam, H., and Lee, D. (2018b). Discovering health benefits of phytochemicals with integrated analysis of the molecular network, chemical properties and ethnopharmacological evidence. *Nutrients* 10**,** 1042.

Yoo, S., Nam, H., and Lee, D. (2018c). Phenotype-oriented network analysis for discovering pharmacological effects of natural compounds. *Scientific reports* 8.
